# Men’s sheds as community-based health promotion for men aged 50 plus: protocol for a mixed-methods systematic review

**DOI:** 10.1186/s13643-021-01762-x

**Published:** 2021-08-04

**Authors:** Birte Marie Albrecht, Linda Foettinger, Karin Bammann

**Affiliations:** Institute for Public Health and Nursing Sciences (IPP), University of Bremen, Grazer Straße 2, 28359 Bremen, Germany

**Keywords:** Men’s sheds, Self-rated health, Subjective well-being, Social isolation

## Abstract

**Background:**

Men are less likely to participate in health promotion. One approach to reach men is the concept of men’s sheds. This community-based health promotion concept brings older men together to engage in joint activities. Prior research revealed various health-related effects of men’s sheds, such as benefits for well-being and mental health. To strengthen the current evidence base of men’s sheds, a mixed-methods systematic review analysing the association between participation and self-rated health, subjective well-being, and social isolation will be conducted. Moreover, information on how to successfully implement men’s sheds will be gathered.

**Methods:**

This mixed-methods systematic review will follow the guidelines of the Joanna Briggs Institute (JBI). The databases MEDLINE (via PubMed), Scopus, Web of Science, and OpenGrey and the websites of men’s sheds associations will be searched for publications. Additionally, a hand search in the reference lists of the included publications will be conducted. Qualitative and quantitative studies published in English, German, or French will be considered for inclusion. The quality of the selected studies will be assessed using the JBI critical appraisal checklists. Following the convergent integrated approach, quantitative data will be transformed into textual descriptions, and subsequently combined with data from qualitative studies as well as from the qualitative components of mixed-methods studies in a simultaneous data synthesis.

**Discussion:**

The results of this systematic review will lead to a comprehensive understanding of the current evidence base regarding the effectiveness of men’s sheds. Furthermore, they will provide useful implications for the implementation of men’s sheds.

**Systematic review registration:**

PROSPERO CRD42020219390

**Supplementary Information:**

The online version contains supplementary material available at 10.1186/s13643-021-01762-x.

## Background

In most countries, men significantly differ from women in terms of life expectancy, morbidity, and health-related behaviour [1]. In Germany, the life expectancy of men at birth, for instance, is 5 years lower than that of women [2]. Gender differences can also be observed in participation in health promotion and prevention programmes. Although men are exposed to greater health risks than women, they are less likely to participate in such interventions [3]. This has been attributed to a low level of acceptance as well as the absence of male-oriented communication strategies to adequately address this specific target group [3–5]. Additionally, there is a lack of men-specific interventions to promote health [6].

Men’s sheds are a concept of community-based health promotion for older men, originally developed in Australia. A men’s shed is a non-profit communal institution for men where they can engage in joint activities. They are predominantly set up as easy-to-access public organisations. The range of activities and general conditions differ from shed to shed. However, repairs and woodwork are central, especially for social and charitable purposes [6]. The Australian Men’s Shed Association (AMSA)—a non-profit organisation that functions as a network between Australian sheds—has defined improving well-being and health as major aims of men’s sheds [7].

Previous studies found that older men have an increased risk of social isolation [8]. Due to its negative effects on mental and physical health, interventions should focus on strengthening the social relationships and integration [9]. Since men’s sheds provide various opportunities to socialise and therefore decrease the risk of social isolation, they can make a substantial contribution to existing health promotion concepts [10, 11]. The shoulder-to-shoulder communication in a supportive environment also enables men to talk about sensitive and shameful topics, including diseases or symptoms [10]. Thus, men’s sheds are a valuable intervention to increase the health literacy of the participating men. Beyond that, men’s sheds are often accompanied by specific events concerning health-related topics, such as diabetes or Alzheimer’s. AMSA, for instance, has designed a specific health programme, which aims to encourage older men to participate in prevention programmes as well as to raise awareness regarding their individual health by using a gender- and age-specific approach [12].

To this date, two scoping reviews [13, 14] and one narrative review [11] on the health-related effects of community-based men’s sheds have been published. The findings are mainly based on qualitative research and are similar across all three publications. There is some evidence that participation in men’s sheds has a beneficial effect on well-being and mental health [11, 13, 14]. According to Milligan et al. [13], this is due to a greater sense of belonging and purpose in life, as well as higher self-esteem and sense of camaraderie. However, the evidence base regarding a potential effect on physical health is insufficient and no conclusions were drawn [11, 13, 14]. To gain a comprehensive understanding of the current evidence base, a mixed-methods systematic review which includes all relevant quantitative, qualitative, and mixed-methods studies is required.

### Objective and research questions

The aim of this research project is to strengthen the evidence base of men’s sheds by analysing their effectiveness and to gather sufficient information regarding a successful implementation of men’s sheds in Germany. It will be guided by the following main research question:

Is participation in community-based men’s sheds associated with (a) self-rated health, (b) subjective well-being, and (c) social isolation in older men aged 50 years and older?

Since there is little practical experience with the implementation of community-based men’s sheds in Germany, additional findings regarding a possible transfer of the concept will be included. Therefore, the systematic review also aims to answer the following further research questions:

Which subgroups of older men participate in men’s sheds or their individual components and which do not?

What general conditions (e.g. opening hours) exist and what influence do they have on participation? Which potentially undesirable effects are reported and how can they be prevented?

What are the characteristics of a successful men’s shed in terms of participation and sustainability? Are they, for instance, equally successful in urban and rural areas?

## Methods

This systematic review protocol follows the reporting guidelines of the Preferred Reporting Items for Systematic Reviews and Meta-Analyses Protocols (PRISMA-P) [15] statement (see checklist in Additional file 1). The systematic review is registered at the International Prospective Register of Systematic Reviews (PROSPERO; CRD42020219390).

The systematic review will be conducted following the guidelines for mixed-methods systematic reviews of the Joanna Briggs Institute (JBI) [16]. We will use a broader definition of quantitative research (standardised data) and qualitative research (unstandardised or semi-standardised data), including not only studies on experience and effectiveness as mentioned in the JBI guidelines, but also descriptive, aetiological, and prognostic studies, as well as mixed-methods studies.

As review software, the System for the Unified Management, Assessment and Review of Information (SUMARI) will be used. SUMARI is a web application developed by JBI which guides the authors throughout the entire systematic review process, enabling effective and efficient collaboration [17].

### Eligibility criteria

#### Study designs

Qualitative, quantitative, and mixed-methods studies will be included in the mixed-methods systematic review. There are no restrictions in regard to the study design, e.g. both observational and interventional studies will be included.

#### Participants

The systematic review focuses on older men aged 50 years and above. Therefore, studies will be included if a minimum of 50% of the study population are at least 50 years old or if separate results for the target population are described.

#### Interventions

Studies will be included if they investigate complex community-based interventions that explicitly refer to the concept of community-based men’s sheds. In this context, ‘complex’ refers to the several interacting components within the men’s sheds, such as activities, health promotion programmes, or informal conversations among the participants [18]. A men’s shed is a public non-profit communal institution for men where they can engage in joint activities. Studies on other community-based activities by men such as voluntary fire brigades in rural areas or men’s regulars will not be included.

#### Comparators

Men who do not participate in men’s sheds serve as the control group.

#### Outcomes

The primary outcomes of the systematic review are (a) self-rated health, (b) subjective well-being, and (c) social isolation.

Self-rated health (a) is a frequently used parameter in epidemiology and public health [19]. A typical measuring instrument is a single question from the 36-item Short Form Survey [20]. It has a high content validity and shows a high correlation with morbidity parameters such as the presence of physical complaints or the number of chronic diseases [21].

Subjective well-being (b) is also a very well-established construct of health research. A widely used instrument is the 5-item questionnaire of the World Health Organization [22], which has a high clinical validity [23] and good test–retest reliability [24].

The multidimensional construct of social isolation (c) is measured differently in quantitative studies and has also been investigated under various terms (i.e. ‘social isolation’, ‘lack of social network’, ‘loneliness’) [8]. Therefore, an assessment of validity and reliability must remain exemplary at this point and can only be examined more closely based on the studies included. A frequently used scale is the Lubben Social Network Scale (LSNS), which has been translated into several languages and adapted to cultural needs. The LSNS showed high validity in a 3-country sample of older adults [25].

Secondary outcomes include the characteristics of men who participate in men’s sheds and of those who do not, the influence of general conditions of men’s sheds on participation, and the characteristics of successful men’s sheds in terms of participation and sustainability.

#### Language

Studies published in English, German, or French will be included in the review.

### Search strategy

The databases MEDLINE (via PubMed), Scopus, and Web of Science will be searched in January 2021 for potentially relevant studies. The search term will focus on the intervention. Further restrictions are not necessary as studies on men’s sheds are still scarce. The piloted search terms can be found in Additional file 2. There are no restrictions in regard to the publication date. Moreover, the database OpenGrey and the websites of the men’s sheds associations will be searched for further relevant publications including grey literature. A hand search in the reference lists of the included publications will complete the search. Since several of the websites of the men’s shed associations provide information about ongoing research projects, the authors will not contact experts for information in this respect.

### Study selection

Results of the search will be checked for duplicates using the reference management programme Citavi. After deduplication, the remaining studies will be exported to JBI SUMARI. First, two authors (LF, BMA) will independently screen the studies by titles and abstracts. Discrepancies are solved by consensus procedures or by a third author (KB). Next, full texts of potentially relevant studies will be reviewed by two independent authors (LF, BMA). Any discrepancies are also solved by consensus procedures or by a third author (KB). Inter-rater agreement will be calculated in SUMARI. The study selection process will be reported in a flow chart displaying detailed information of the studies included and excluded in each step.

### Quality assessment

The JBI critical appraisal checklists [16] will be used for the assessment of the methodological quality of the selected studies. Quantitative and qualitative studies will be critically assessed with seven different checklists (one qualitative, six quantitative) depending on their study design provided by JBI. The checklists are accompanied each by an extensive tool guidance containing the judgement standard for each checklist item [26].

The checklist for qualitative studies includes questions to determine the congruity between philosophical perspective and methodology as well as the congruity between methodology and research question, method of data collection and analysis, and representation of data, respectively. Furthermore, the cultural or theoretical background of the researcher, the influence of the researcher on the research, adequate representation of the participants, ethical approval, and the conclusions drawn from the data are addressed [27]. Checklists for quantitative research comprise cross-sectional studies, case–control studies, cohort studies, prevalence studies, quasi-experimental, and randomised controlled trials. As an example for the quality assessment of quantitative studies, the checklist for cross-sectional studies includes questions on the definition of inclusion criteria; description of study subjects and setting; measurement of exposure, condition, and outcomes; identification of and dealing with confounding factors; and appropriateness of statistical analysis [28]. For mixed-methods studies, all applicable checklists will be applied.

For the quality assessment, the questions in the checklists must be answered with yes, no, unclear, or not applicable. Since the overall methodological quality of the studies will have no influence on their inclusion in the systematic review, we decided not to devise a weighing scheme for calculating an overall quality score, but to conduct the quality assessment differentiated by single items.

Two authors (LF, BMA) will conduct the assessment independently. Discrepancies are solved with the involvement of a third author (KB) by consensus procedures. Agreement between reviewers will be assessed for each category by intra-class correlation coefficient.

### Data extraction

Data of the included studies will be extracted by two authors (LF, BMA) independently. Discrepancies are solved by consensus procedures or by a third person (KB). The standardised JBI tool will be used for data extraction [16]. This will be modified and extended in conjunction with health promotion practitioners. The following information will be extracted: publication details, study design, participants’ characteristics, intervention details, details of the control group, and primary and secondary outcomes. Original authors will be contacted if further non-published data is needed. If multiple papers from the same study are published, information and results of both papers will be collated.

### Data transformation

In our mixed-methods review, we follow a convergent integrated approach, which involves integration of transformed data. Therefore, two authors (LF, BMA) will convert data extracted from quantitative studies as well as from the quantitative components of mixed-methods studies into textual descriptions by conducting a narrative interpretation of these quantitative data (‘qualitizing’).

### Data synthesis

Qualitized data will be combined with the data extracted directly from qualitative studies as well as from the qualitative components of mixed-methods studies. Subsequently, this combined data will be examined and coded by two authors (LF, BMA) on the basis of similarity in meaning. By grouping the emerged codes, categories will be identified. To produce the overall integrated findings of the review, these categories will be aggregated.

### Reporting of results

Results will be reported using the Preferred Reporting Items for Systematic Reviews and Meta-Analyses (PRISMA) guideline [29]. The characteristics of all included studies (author/s, year, country, study design, intervention details, study population details) will be displayed in a tabular summary. The categories emerging from the data synthesis will be presented in a separate table with corresponding examples.

## Discussion

The objective of the mixed-methods systematic review is to analyse the effectiveness of men’s sheds with regard to self-rated health, subjective well-being, and social isolation. Moreover, we want to gather information on how to successfully implement men’s sheds in Germany.

Older men are a rather neglected group in health promotion [5]. Although significant sex differences in regard to life expectancy, morbidity, and health-related behaviour exist [1], there is a lack of men-specific health promotion interventions [6]. The community-based approach of men’s sheds could fill this gap. Over the course of the last years, several studies investigating different aspects of men’s sheds have been published (e.g. [30–32]). Synthesising the results from this wide spectrum of publications will strengthen the evidence of the potential impact of men’s sheds on men’s health and will moreover summarise important practice-related information available on this concept. The mixed-methods approach of the systematic review includes both quantitative and qualitative publications and will, therefore, provide a comprehensive overview of the published findings.

A limitation of the systematic review is that only studies published in English, German, or French will be included. However, we do not expect this to bias results as the concept of men’s sheds originated in English-speaking countries, and sheds seem to be most prominent in these countries.

If the results regarding the effectiveness of men’s sheds are promising, the review could encourage other countries to consider men’s sheds for the promotion of men’s health. Furthermore, the results of the review will provide useful implications for the implementation process.

## Supplementary Information

**Additional file 1:** PRISMA-P 2015 Checklist

**Additional file 2:** Piloted search strategy

## Data Availability

Not applicable.

## Abbreviations

AMSA: Australian Men’s Shed Association
JBI: Joanna Briggs Institute
LSNS: Lubben Social Network Scale
PRISMA: Preferred Reporting Items for Systematic Reviews and Meta-Analyses
PRISMA-P: Preferred Reporting Items for Systematic Reviews and Meta-Analyses Protocols
PROSPERO: International Prospective Register of Systematic Reviews
SUMARI: System for the Unified Management, Assessment and Review of Information
