# Supplementary material for: Men’s sheds as community-based health promotion for men aged 50 plus: protocol for a mixed-methods systematic review
Source: Syst Rev. 2021 Aug 4;10:215. doi: 10.1186/s13643-021-01762-x (PMC8336413; doi:10.1186/s13643-021-01762-x)
Supplement: Supplementary file 2 — Additional file 2: Piloted search strategy [file 13643_2021_1762_MOESM2_ESM.docx]

**Piloted search query:**

| **Database** | **Search terms** |
| --- | --- |
| Medline (via PubMed) | ("Men's shed*") OR ("Men in sheds") |
| Web of Science | TS=("Men's shed*" OR "Men in shed*") |
| Scopus | TITLE-ABS-KEY("Men's shed*" OR "Men in shed*") |
| OpenGrey | "men* shed*" |
